# Motor Behavior Regulation of Rat Robots Using Integrated Electrodes Stimulated by Micro-Nervous System

**DOI:** 10.3390/mi15050587

**Published:** 2024-04-28

**Authors:** Jiabing Huo, Le Zhang, Xiangyu Luo, Yongkang Rao, Peili Cao, Xiaojuan Hou, Jian He, Jiliang Mu, Wenping Geng, Haoran Cui, Rui Cheng, Xiujian Chou

**Affiliations:** 1 Science and Technology on Electronic Test and Measurement Laboratory, North University of China, Taiyuan 030051, China; 2 Fifth Clinical Medical School, Shanxi Medical University, Taiyuan 030012, China; 3 Department of Neurosurgery, Shanxi Provincial People’s Hospital, Taiyuan 030012, China

**Keywords:** micro-nervous system, integrated electrodes, rat robots, motor behavior regulation

## Abstract

As a cutting-edge technology, animal robots based on living organisms are being extensively studied, with potential for diverse applications in the fields of neuroscience, national security, and civil rescue. However, it remains a significant challenge to reliably control the animal robots with the objective of protecting their long-term survival, and this has seriously hindered their practical implementation. To address this issue, this work explored the use of a bio-friendly neurostimulation system that includes integrated stimulation electrodes together with a remote wireless stimulation circuit to control the moving behavior of rat robots. The integrated electrodes were implanted simultaneously in four stimulation sites, including the medial forebrain bundle (MFB) and primary somatosensory cortex, barrel field (S1BF). The control system was able to provide flexibility in adjusting the following four stimulation parameters: waveform, amplitude, frequency, and duration time. The optimized parameters facilitated the successful control of the rat’s locomotion, including forward movement and left and right turns. After training for a few cycles, the rat robots could be guided along a designated route to complete the given mission in a maze. Moreover, it was found that the rat robots could survive for more than 20 days with the control system implanted. These findings will ensure the sustained and reliable operation of the rat robots, laying a robust foundation for advances in animal robot regulation technology.

## 1. Introduction

The brain-computer interface (BCI) is a rapidly developing technology that establishes a bidirectional communication channel between the brain and external electronic devices. In the medical field, BCI technology has shown significant promise in treating a range of neurological disorders, including stroke [1], epilepsy [2], depression [3], and movement disorders [4,5,6]. Another promising field for BCI application is to control animal robots using a write-in BCI approach [7]. The specific aspects of this approach lie in the application of an electrical signal into determined regions of the animal’s brain so that neuronal activity is stimulated in the corresponding areas to control the animal’s locomotor behavior and aid in performing challenging or hazardous tasks for humans. Through the combination of the distinct advantages of animals and traditional bionic robots, animal robots exhibit more obvious advantages in terms of environmental adaptability and dexterity. Inspired by this methodology, significant successful studies have been conducted on various animal robots, such as rats [8,9], pigeons [10,11], geckos, insects [12], and other diverse species [13,14].

At the moment, the main stimulation methods for controlling animal robots include ultrasonic stimulation [15], light stimulation [16,17], and electrical stimulation. Ultrasonic stimulation is a non-invasive modulation technique that modifies the neuronal activity in the brain using pulsed ultrasound waves with specific parameters. A previous study reported that specific ultrasonic waves were applied to stimulate a particular region in a macaque monkey’s brain, influencing its decision to choose a target. However, the size of the ultrasonic device restricts its use to long-distance transmission, which is only suitable for laboratory settings after restraining the animal. The most commonly used method of optical stimulation is optogenetic stimulation, which involves synthesizing light-sensitive proteins through implanted viral vectors in the brain and using different wavelengths to modulate neural function through light stimulation [18]. Previous studies have demonstrated that the optogenetic stimulation of the dIPAG nucleus in rats allowed for the modulation of robotic resting behavior [19]. However, the adaptability of viral vectors and the interference from response signals of photosensitive proteins create very significant obstacles for their application. Electrical stimulation is the primary method for controlling the movement of animal robots, which can achieve behavioral control through the stimulation of relevant motor brain areas, facial whiskers, and motion-related muscles [20]. Electrical stimulation is characterized by stability, adjustable parameters, and high efficiency, throwing light on the control of locomotor behavior in animal robots. However, only a single wave was applied in most previous electrical stimulation cases, and a comparison of the effects of different stimulus waveforms is lacking. It is necessary to develop a wireless voltage stimulation device that can generate multiple stimulation waveforms.

Stimulating electrodes are an essential factor in the success of animal robots. Currently, the microelectrodes used in research mainly include fiber-optic [16], glass [21], and metal microfilament electrodes. Fiber-optic microelectrodes can only be appropriate for modulating neural activity through light-based stimuli, such as optogenetics. In a previous study, it was revealed that glutamatergic neurons in the lateral paragigantocellular nucleus (LPGi) were capable of regulating locomotor speed based on the intensity of light stimulation [22]. Glass microelectrodes are simple to fabricate and suitable for short-term stimulation and recording of cell potentials. However, it is a critical task to meet the requirements for long-term electrical stimulation experiments for glass microelectrodes. Due to their excellent biocompatibility and compliance with resistance and toughness requirements, metal microelectrodes are widely applied in animal robotics research. For rat robots, the electrical stimulation sites investigated mainly encompass the MFB, which is used to elicit virtual rewards and propel rats forward [23], and the SIBF, ventral posteromedial nucleus (VPL), ventral posteromedial nucleus (VPM) [24], and nigrostriatal pathway (NSP) [25,26], which are utilized for left–right turning control. Therefore, the implantation of electrodes is required at multiple stimulation sites. Currently, unit-point stimulation electrodes are frequently used in animal robots, which increase the surgical difficulty if multiple sites need to be implanted at the same time and cause a great deal of uncertainty. Secondly, the solidity of the unit-point electrode is poor. After surgery, rats will often scratch the electrodes due to the foreign body sensation in the brain, which renders the electrodes prone to dislodgement, leading to postoperative infections and other problems.

Given the existing research gap, an integrated stimulation electrode stimulating the MFB and S1BF of rats simultaneously using a radio-stimulation stimulate bag to control rat locomotion is systematically studied in this manuscript. The integrated electrodes are beneficial for simplifying the surgical implantation process. The integrated electrodes can also effectively prevent the phenomenon of electrode dislodgement after surgery and avoid secondary injury to the rat, thus enabling a longer survival time for the rat robots. In this study, the neurostimulation module can generate three types of waveforms, including sine, triangle, and square, which are used to compare the stimulation effect of different waveforms. Eventually, we succeeded in controlling the forward and steering motor behaviors of the rats and manipulating the rats to perform complex maze tasks. The rat robots in this study have contributed to disaster relief and intelligence gathering, laying a solid foundation for the application of animal robotics in military and civilian rescue.

## 2. Materials and Methods

### 2.1. Subjects and Ethical Statement

In this study, 8- to 10-week-old adult male Sprague-Dawley (SD) rats weighing 300–350 g were used due to their significant disease-resistant profile and being reproductively active, which endows them with massive potential for use in animal robots. All procedures were approved by the Ethics Committee Review Board of the North University of China (20221103). The number of experimental animals was reduced as much as possible based on the necessity of using animals in experiments.

### 2.2. Integrated Electrode

**Figure 1:**
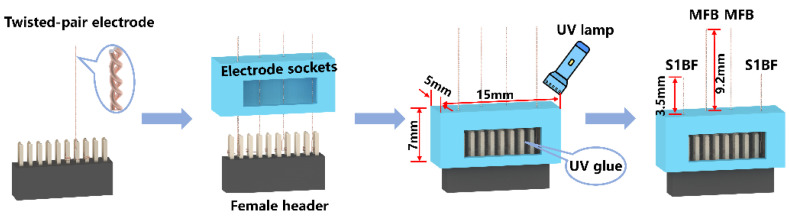
Flow chart of integrated electrode preparation.

The electrical stimulation sites of the rat robot involved two brain regions, including the cerebral cortex and deeper parts of the brain. To reduce surgical complexities and enhance electrode implantation accuracy, integrated electrodes that were capable of simultaneous implantation across multiple sites were employed in this study, which greatly improved stability and biocompatibility and minimized the risk of the rats grasping or snapping at the electrodes. The material used for the electrode wire was a nickel–titanium alloy electrode wire with an inner diameter of 50 μm and an insulating layer of 8 μm thick. In this study, the integrated electrodes were divided into two components: a dual-channel cortical electrode for stimulating the S1BF and a dual-channel deep electrode for stimulating the MFB. The primary production process of the integrated electrodes was as follows (Figure 1):

(1) Two electrode wires of appropriate length were twisted together using a twisting device to ensure sufficient strength when piercing the cerebral cortex. Four twisted-pair Ni-Ti electrodes were manufactured, with 1 cm of insulation removed from the tails, and the two electrode wires at the end of the twisted pair were wrapped around the 1.27 mm pitch row mother.
(2) Holes were drilled in the electrode sockets (length of 15 mm, width of 5 mm, height of 7 mm) following the mediolateral (ML) and anteroposterior (AP) coordinates of the implantation site, and then the electrode was passed through the hole in the electrode base.
(3) A UV curing adhesive was injected into the electrode base, and the position of the electrode wire was adjusted before irradiation with a UV lamp for 5–10 s to secure it.
(4) The electrodes were diagonally cut according to the dorsoventral (DV) coordinates to prevent short-circuiting of the electrodes. Additionally, a 1 mm margin in length was retained for the electrodes, accounting for the thickness of the skull itself. The final electrode length was 9.2 mm for the MFB site and 3.5 mm for the SIBF site.

### 2.3. Animal Experiment

Rats of an appropriate weight were selected, and the whole electrode implantation procedure was completed in a small animal anesthesia machine. Anesthesia was induced via the administration of a mixture of air (flow rate: 3 L/min) and isoflurane (concentration: 3%), and anesthesia was maintained with isoflurane at a concentration of 2.2–2.5%. The absence of a leg contraction response confirmed that anesthesia had been successfully applied to the rats. Then the rat’s hair on the head was shaved and fixed on the stereotaxic apparatus, accompanied by local disinfection on the head surface using iodophor. Following that, an appropriate amount of lidocaine (10 mg/mL) was injected subcutaneously, representing surface analgesia. An elliptical trauma was created by cutting the epidermis and subcutaneous tissues appropriately from the back of the rat’s eye corners to the line between the ears, exposing the bony sutures on the cranial surface and the area between the fontanelles (Bregma) and the tip of the herringbone suture (Lambda). The height of these two points was adjusted to maintain an anterior–posterior level of the cranial surface. Heating pads were used to maintain the body temperature of the rats during surgery. The stimulation target sites selected in this study were provided with the following coordinates:

MFB (couple): AP: −3.8 mm, ML: ±1.6 mm, DV: −8.2 mm;

S1BF (couple): AP: −1.8 mm, ML: ±5.0 mm, DV: −2.5 mm.

After the electrode implantation sites on the skull were marked, 3 to 4 small holes were drilled laterally using a cranial drill to anchor the cranial nails. The use of integrated stimulation electrodes ensured efficient electrode implantation and improved the accuracy of the procedure. After electrode implantation, dental cement was applied evenly to the incision site. When the dental cement hardened and the incisions were sterilized again with iodophor, the rat was safely removed from the stereotaxic apparatus.

### 2.4. Remote Control System for Rat Robots

**Figure 2:**
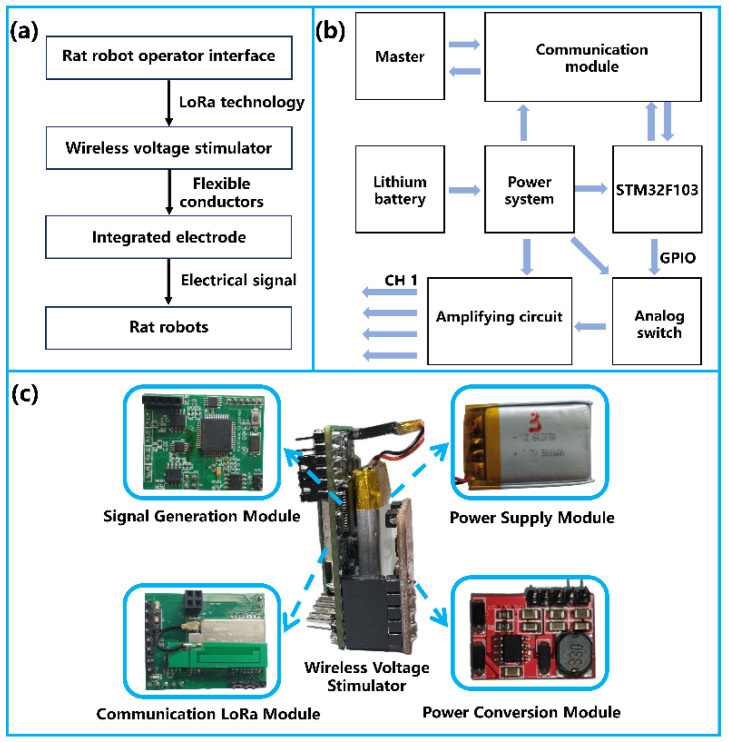
Rat robotic stimulation system: (**a**) neurostimulation system framework; (**b**) working frame of the wireless voltage stimulator; (**c**) wireless voltage stimulator.

Figure 2a illustrates the neurostimulation system framework used in this study. The rat robot operator interface and the wireless voltage stimulator communicate data bidirectionally via LoRa technology, and the wireless voltage stimulator is connected to the integrated electrodes with flexible wires. Finally, the electrical signals are fed into the corresponding channel brain area of the rats. The working framework of the wireless voltage stimulator is shown in Figure 2b. The stimulator consists of a signal generation module, a power supply module, a communication LoRa module, and flexible leads. In the stimulator, the LoRa module receives stimulation parameters (channel configuration, waveform, amplitude, frequency, and duration) from the console and facilitates communication over a distance of about 3 km, surpassing other modules with equivalent power conditions in terms of transmission range. The microcontroller STM32F103RCT6 (STMicroelectronics, Geneva, Switzerland) performs digital-to-analog conversion of the received data and generates stimulation waveforms for the respective channels. The stimulator is powered by a rechargeable lithium battery (3.7 V, 300 mAh) and is connected to the integrated electrodes implanted in the rat’s brain via flexible leads. The physical object of the wireless voltage stimulator is shown in Figure 2c.

### 2.5. Electrical Stimulation Experiments

The direct stimulation of the MFB for untrained rat robots is supposed to lead to head-lifting and accelerate whisker-twitching behaviors, which could not be directly correlated with forward locomotion. Similarly, stimulating the ipsilateral S1BF of rats caused them to deflect to the same side. However, the direct electrical stimulation of the SIBF in untrained rats resulted in uncertainty in their left–right turning behavior. Therefore, a training process is required to control the forward and steering movements of the rat robots. Before the training process, a range of parameters, such as waveforms, amplitudes, frequencies, and duration times of electrical stimulation, were assessed on a rat robot employing a radio-voltage stimulator. Optimal training parameters were determined based on observations of the head-raising and accelerated whisker-twitching behaviors of the rat robots. Then, the rats were subjected to immobilized reinforcement training in a circular open field and an eight-arm radial maze. The training lasted one to two weeks, which was beneficial for enabling the rats to establish an innate relationship between electrical stimulation and motor behavior. In animal behavioral experiments, it is essential to provide a quantitative analysis of the animal’s behavior at every moment. To achieve real-time tracking of rat movement trajectories, a real-time motion capture system was built using a camera and behavioral analysis software.

### 2.6. Histological Evaluation

Due to slight differences in brain size among rats, there may be deviations in the electrode implantation position. To assess the accuracy of electrode implantation, the rats were euthanized post-experiment, and the brains were sectioned and stained with hematoxylin and eosin (H&E) stains to observe the electrode position. The MFB area, due to its positioning deep within the brain and relatively small size, requires particular attention during the assessment process.

## 3. Results

### 3.1. Integrated Electrodes and Backpack

**Figure 3:**
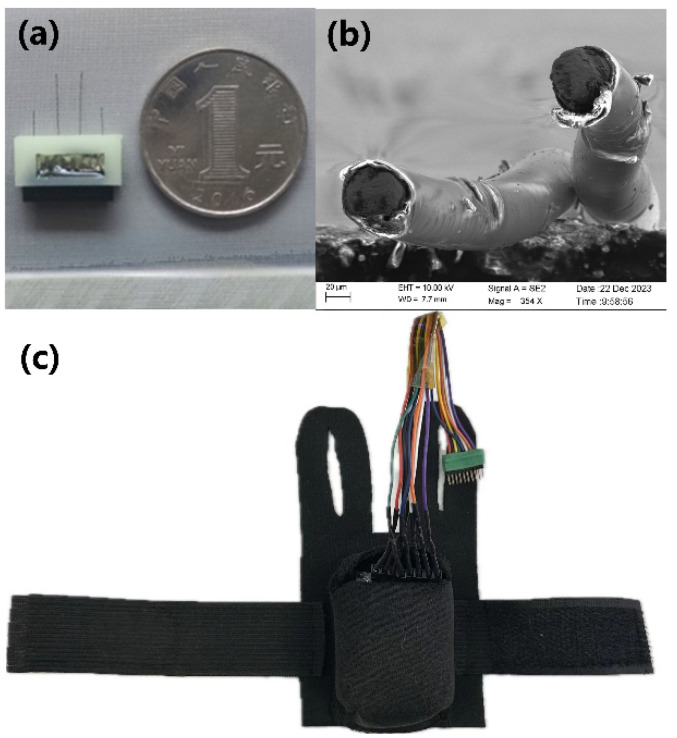
Integrated electrode analysis: (**a**) physical view of the integrated electrodes; (**b**) SEM electron microscopy of twisted wire electrodes; (**c**) rat robot backpack.

Figure 3 shows the physical view of the integrated electrodes (a) and SEM electron microscopy (b) of the twisted-pair Ni-Ti electrodes. A well-preserved insulation of the metal electrode wires with the split at the top is displayed, which ensures that the electrodes will not be shorted out. The rat robot backpack is shown in Figure 3c; this secures the stimulation module to the rat and connects the stimulation module to the electrodes.

**Figure 4:**
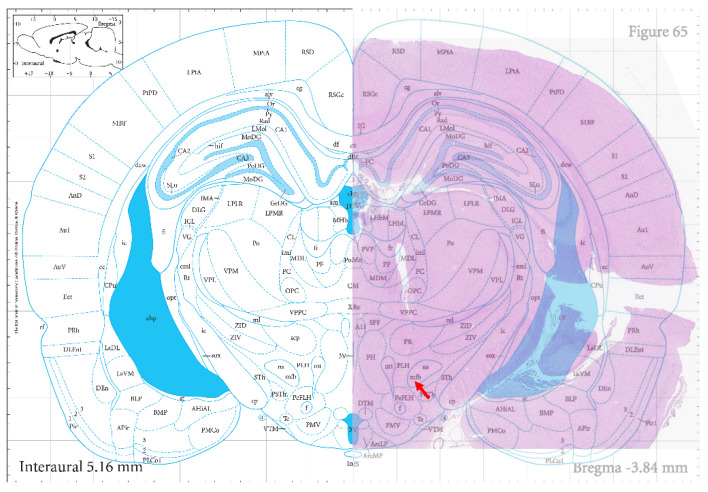
Hematoxylin–eosin staining results.

During the electrode implantation procedure, it was observed that the duration for surgical implantation of the integrated electrodes was reduced by about half compared to the previously utilized single electrodes due to only one implantation site requiring correct localization, and then it was possible to follow naturally and precisely with the other sites. The real-time monitoring of the rats’ postoperative condition revealed that they exhibited stable and healthy survival for over 20 days (Video S1, Supplementary Materials), demonstrating successful adaptation to the integrated electrodes. The hematoxylin–eosin stains of the rat coronal brain sections are illustrated in Figure 4, and the arrows are the locations of MFB. The red area shows the actual results of staining the rat brain sections, which clearly highlights the location of the medial forebrain bundle electrode implantation pathway compared to the standard rat brain atlas at the bottom. Some neurons around the electrode paths were also disarranged, and some inflammatory cells were presented, as shown in Figure S1 in the Supplementary Materials.

### 3.2. Activation Parameters of Rat Robotic Brain Regions

It is necessary to determine the appropriate electrical stimulation parameters before the rat robot can be trained efficiently, such as waveform, amplitude, frequency, and duration at various stimulation sites. Selecting a fully functional rat robot and determining the optimal stimulation parameters for subsequent training were the goals of this element of the experiment. The brain area activation value is the electrical stimulation parameter that elicits a significant response in rats. The current electrical stimulation was considered effective if the rats showed obvious head-up or whisker-accelerated twitching behavior under MFB stimulation and head-turning and whisker-accelerated twitching behavior under SIBF electrical stimulation. After waiting for the rats to be completely calm, the corresponding brain regions of the rat robots were stimulated again using the same parameters, and the stimulation parameters were considered reasonable if there was still a corresponding response. The MFB and S1BF brain region activation values in six rats (S-1, S-2, S-3, S-4, S-5, and S-6) were examined in this study, with the detailed data presented in the Supplementary Materials (Tables S1–S6).

The findings revealed that among the four brain regions tested in the same rat, square waveforms required a lower amplitude, frequency, and duration than sine and triangle waveforms. Therefore, it can use lower stimulation parameters to achieve the appropriate stimulation effect and reduce brain damage in the rat robot, indicating the greater efficiency of the square waveforms for stimulation. Despite the same rat type and similar body weights being employed, it should be noted that individual differences in rats resulted in considerable variability in the activation values of brain regions. The stimulus parameters outlined in Table 1 were utilized for subsequent training experiments with rats named from S-1 to S-6. It was found that the amplitude between 0.6 V and 2.1 V, the frequency between 120 Hz and 180 Hz, and the duration time between 0.4 and 0.6 s were suitable for the stimulation parameters of MFB. The amplitude between 1.5 V and 4.2 V, the frequency between 110 Hz and 250 Hz, and the duration time between 0.3 and 0.7 s were suited for the stimulation parameters of SIBF.

### 3.3. Maze Training

**Figure 5:**
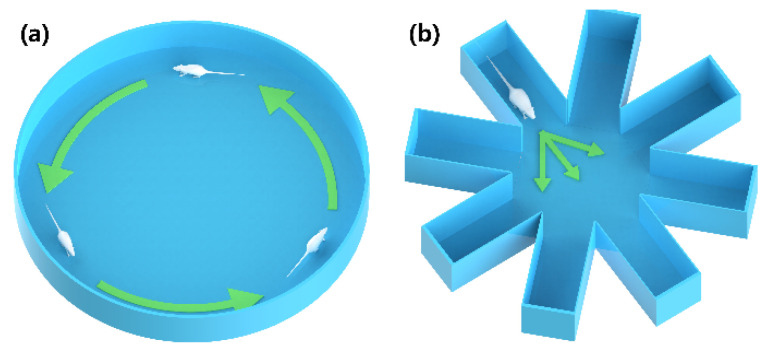
Training grounds for rat robots: (**a**) circular open field; (**b**) eight-arm radial maze.

The circular open field with a diameter of 1 m, as depicted in Figure 5a, was utilized to train rats to move forward. Rats have a natural tendency to hug the walls or seek refuge in the corners of an open field, which hinders subsequent forward training in a conventional square open field. The circular field has the advantage over the square field of eliminating blind corners, which obviate the concern of rats hiding or becoming stuck in the corners. The rats equipped with the backpacks were placed on the surface of the circular open field, and appropriately intense MFB electrical stimulation was administered once the rats commenced forward movement, which will induce continuous forward locomotion. The reward-based electrical stimulation was stopped when the rat stopped moving forward, and the same operation was conducted for the forward movement, which could be used to establish the link between forward locomotion and MFB reward stimulation. The training was completed when the rat completed a lap of walking in the circular open field. After each training session, the rats were given a ten-minute rest in order to return to a calm state. Five to ten training sessions were conducted daily for one to two weeks. Following successful training, it was observed that stimulation of the MFB brain region could induce forward movement in stationary rat robots and accelerate rats already in motion.

An eight-arm radial maze with a 40 cm arm length, as presented in Figure 5b, was then employed to train rats in steering movements. The rat was placed in one arm of the maze, and continuous steering cue stimulation was applied to the S1BF site when the rats arrived at the central path selection area of the maze. If the rat’s head turned in a predetermined direction, the MFB-rewarding stimulus was promptly administered; otherwise, moderate punishment via physical means was applied. MFB stimulation is used as a reward to induce the movement of resting rats. Alternatively, physical stimuli such as pinpricks and electric shocks can punish or prompt the rats to move. Five steering training sessions were defined as a set of experiments, and rats were allowed to rest for a period of time after each set of experiments. Five sets of experiments were conducted daily for one to two weeks until the rats were able to steer quickly and successfully on command. Improvements in steering command responsiveness were demonstrated in rats compared to pre-training. After two weeks without any electrical stimulation, the rats could still execute the corresponding turn actions upon restimulation, indicating the enduring efficacy of the training.

### 3.4. The Cube Maze Mission

**Figure 6:**
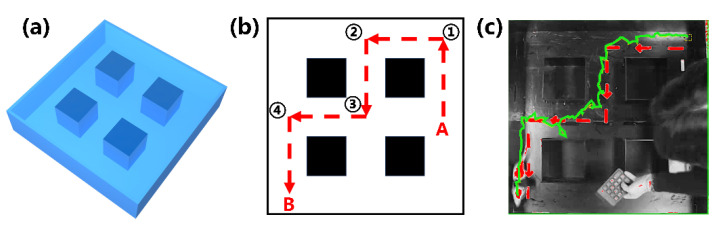
Rat robot performs tasks in the cube maze: (**a**) Rubik’s cube maze model; (**b**) task execution path; (**c**) trajectory diagram of rats performing the task.

In Figure 6a, the 1 m × 1 m cube maze is shown. This was constructed to simulate a task scenario for the rat robot. Rat robots successfully trained in a circular open field and eight-armed radial mazes were deployed in the cube maze to execute tasks. During the experiment, rats started at point A and then sequentially passed along the target path, shown with a dash line in Figure 6b, and reached point B, which was defined as success. A real-time motion capture system was built to analyze the rat movement trajectories. In Figure 6c, the red arrows are the preset paths, and the greener route is the actual trajectory of the rat robot. The rat successfully followed the designed route to reach the destination (Video S2, Supplement Materials).

**Figure 7:**
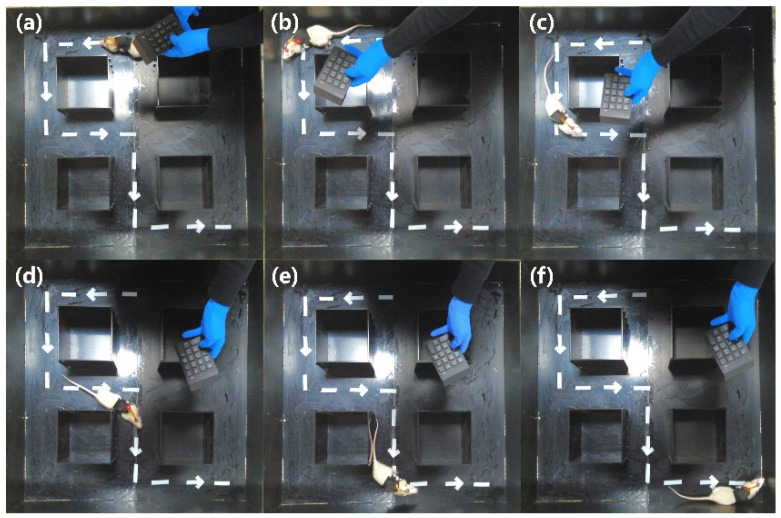
Rat robot steering in decision areas: (**a**) the starting position; (**b**) turning behavior at decision point 1; (**c**) turning behavior at decision point 2; (**d**) turning behavior at decision point 3; (**e**) turning behavior at decision point 4; (**f**) the finishing position.

The steering behavior of the rat robot within the cube maze shown in Figure 6a is further illustrated in Figure 7, presenting a “left-right-left” orientation pattern. To ensure the successful entry of the rats into the preset channel, multiple electrical stimulation commands were employed when the rats reached the decision points, such as points 1, 2, 3, and 4, given in Figure 6b. When the rat arrived at decision points 1, 2, and 4, the left-turn command was initiated. When the rat arrived at decision point 3, a right-turn command was given. It can be seen that, after training, the rat could accomplish the mission of moving forward along the given path.

**Figure 8:**
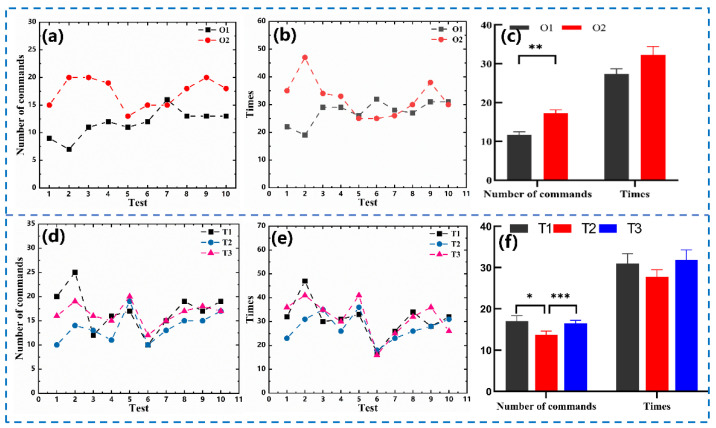
Behavioral analysis of rat robots in different states: (**a**) number of commands required to complete O1 and O2; (**b**) time required to complete O1 and O2; (**c**) a comparison of O1 and O2 command numbers and times; (**d**) number of commands required to complete T1, T2, and T3; (**e**) time required to complete T1, T2, and T3; (**f**) a comparison of T1, T2, and T3 command numbers and times. * *p* < 0.05, ** *p* < 0.01, *** *p* < 0.001.

In terms of the practical application of rat robots, it is necessary to quantify the number of commands (Figure 8a) and time (Figure 8b) required for completing the maze tasks. After one week of successful training, the rats were trained without any training and were directly allowed to perform the maze task (O1). After a good amount of time to rest again, the rats were trained again for three days, controlling them to perform the maze task (O2). A half-hour interval between each task was used to ensure that the rats returned to their normal state for six to eight hours per day. Results showed that the number of commands required to complete O2 (17.3 ± 0.82) were greater than those of O1 (11.7 ± 0.78), which is also applicable for the time needed to complete the same mission, specifically for O2 (32.3 ± 2.15 s) and O1 (27.4 ± 1.31 s). A significant difference in the number of commands and time for O1 and O2, shown in Figure 8c, suggests that prolonged training may affect the state of the rats. In Figure 8d,e, the number of commands and time needed for completing the maze task consecutively three times (T1, T2, and T3) without any interruptions are displayed. The number of commands and time required for T2 (commands: 13.7 ± 0.93, times: 27.7 ± 1.79 s) were lower than those for T1 (commands: 17 ± 1.33, times: 31 ± 2.36 s), but the number of commands and time taken for T3 (commands: 16.5 ± 0.72, times: 31.8 ± 2.47 s). The comparison of these results, provided in Figure 8f, suggests that the length of stimulation time affects the efficiency of the rat robots.

## 4. Discussion

The stimulation electrode plays a crucial role in ensuring the survival and effectiveness of the control of the animal robot. An integrated electrode that offers customization options for stimulation sites with different coordinates based on specific requirements is presented in this study. As a significant attribute of this design, the surgical time was shortened, and the survival of the rat robot was improved. The reliability of the integrated stimulation electrodes was confirmed through observation of the intact electrode channels in the rat brain during staining.

The integrated electrodes used in this study were initially designed for stimulation, and their function can be extended to serve as acquisition electrodes, therefore integrating both stimulation and acquisition capabilities [27,28]. An acquisition electrode constructed with metal microfilaments can be used to record the action potential spike signals or local field potential (LFP) signs of rats. After applying electrical stimulation to the rat’s brain, the acquisition electrode captures the changes in neuroelectric signals, enabling the analysis of the impact of electrical stimulation on the rat brain’s electrical activity, which will be studied in further work.

Selecting appropriate stimulation waveforms and parameters is critical for neuromodulation in rats, as the effects of different waveform stimulations can vary significantly. In this study, brain activation values using three waveforms were tested in four brain regions of rats. Initially, the stimulation parameters were set at high levels and subsequently reduced the stimulation intensity, frequency, and duration by 0.3 V, 10 Hz, and 0.1 s, respectively. In rats, effective stimulation is indicated by significant head-lifting and whisker-accelerated twitching behavior. Only one parameter is modified for each stimulation, starting with determining the intensity, then the frequency, and concluding with the duration time. The experimental results demonstrated that the stimulation effect of the square wave was superior to that of triangular and sine waves, with lower stimulation parameters required to activate the targeted brain area. Consequently, in this study, square waves were considered more suitable for controlling rat robots. Considering the diversity of waveforms, a broader range of waveforms was planned to be used for assessment of the stimulation effect with minimal amplitude, frequency, and duration time, with the purpose of mitigating brain damage resulting from electrical stimulation in rats.

It was demonstrated that rat movement can be controlled through stimulation of the MFB and the SIBF in rats [29]; however, high-efficiency training should still be pursued. Previous studies of training the MFB of rats using a square open field resulted in the rats naturally spending a long time cowering in the corners. However, using a circular open field can avoid this problem and significantly shorten the training time of the MFB. Unlike previous studies of MFB stimulation, stimulating the MFB using larger parameters may induce the steering effect. This is because the MFB is very close to the nigrostriatal pathway (NSP), and stimulation of the NSP brain region causes the rats to turn. Therefore, the accuracy of electrode implantation in the MFB brain region is also a critical factor in the success of behavioral modulation.

The states of the rats were classified as calm and active, and the stimulation effect varied according to the state. The calm state necessitated more commands or higher electrical stimulation parameters for the rats to respond. In the process of MFB training, rats in the calm state exhibited small amplitudes of head-up or stride movements, whereas rats in the active state demonstrated greater amplitudes of motion. During the cube maze task performed by the rat robots, active rats achieved successful steering with only one or two commands, whereas calm rats necessitated more than three stimulus commands to achieve successful steering. Therefore, it is more effective to train rats when they are active.

Finally, there are still some unavoidable problems with rat robots that we need to continue to explore. To address the issue of energy supply, the researchers implanted a combination of a biofuel cell and a stimulator into pigeons and then powered the stimulator through a glucose redox reaction in the biofuel cell. In addition, solar energy [30] and magnetic induction power supplies [31] have also been tested, yet environmental factors significantly influence their practical utility. Consequently, traditional battery power remains the predominant choice. There are subtle differences in different rat brain regions, such as different levels of impedance in the brain region. In addition, the long-term electrode implantation will inevitably cause an inflammatory response, increasing stimulus sensitivity or tolerance in rats [32]. When stimulating the somatosensory cortex, there may be interference (lag in stimulation, instability) due to the integration of the various sensory input signals in the brain, which will degrade modulation accuracy.

## 5. Conclusions

A novel integrated stimulation electrode and its success in controlling a rat robot through wireless electrical stimulation devices are presented in this study. The results suggest that using integrated electrodes can enhance surgical efficiency and increase the survival rate of rats, providing a solid foundation for the practical application of the rat robot. Furthermore, reasonable training stimulation parameters could not only prevent rats from experiencing irreversible brain damage caused by excessive or rapid electrical stimulation but also improve training effectiveness through enabling rats to relate electrical stimulation with behavior. These findings provide a reliable way to control rat robots and can be expanded to other animal robots.

## Figures and Tables

**Table 1:** Stimulation parameters in rats.

		S-1	S-2	S-3	S-4	S-5	S-6
Left SIBF	Amplitude (V)	2.7	4.2	1.5	2.7	3	4.2
	Frequency (Hz)	180	250	150	170	250	230
	Duration time (s)	0.6	0.7	0.5	0.4	0.7	0.7
Left MFB	Amplitude (V)	2.1	1.5	1.8	1.8	0.6	2.1
	Frequency (Hz)	130	150	150	130	150	180
	Duration time (s)	0.6	0.5	0.4	0.5	0.5	0.5
Right MFB	Amplitude (V)	1.8	2.1	2.4	1.8	1.2	1.8
	Frequency (Hz)	130	170	170	120	150	150
	Duration time (s)	0.5	0.5	0.6	0.4	0.5	0.5
Right SIBF	Amplitude (V)	3.6	3	1.5	3.6	3.3	3.6
	Frequency (Hz)	220	200	110	200	200	250
	Duration time(s)	0.7	0.7	0.3	0.4	0.7	0.7

## Data Availability

The original contributions presented in the study are included in the article, further inquiries can be directed to the corresponding author.

## Supplementary Materials

The following supporting information can be downloaded at https://www.mdpi.com/article/10.3390/mi15050587/s1. Table S1: Stimulation parameters in S-1 rat. Table S2: Stimulation parameters in S-2 rat. Table S3: Stimulation parameters in S-3 rat. Table S4: Stimulation parameters in S-4 rat. Table S5: Stimulation parameters in S-5 rat. Table S6: Stimulation parameters in S-6 rat. Figure S1: Local enlargement of staining results of rat coronal brain sections. Video S1: Rats surviving more than 20 days after electrode implantation. Video S2: A signal maze task performed by a rat robot.
