# Supplementary material for: Motor Behavior Regulation of Rat Robots Using Integrated Electrodes Stimulated by Micro-Nervous System
Source: Micromachines (Basel). 2024 Apr 28;15(5):587. doi: 10.3390/mi15050587 (PMC11123150; doi:10.3390/mi15050587)
Supplement: Supplementary file 1 [file micromachines-15-00587-s001.zip › Supporting Information.pdf]

Motor Behavior Regulation of Rat Robots Using Integrated Electrodes Stimulated by Micro-Nervous System

Jiabing Huo [1], Le Zhang [1,*], Xiangyu Luo [1], Yongkang Rao [1], Peili Cao [2,3], Xiaojuan Hou [1,*], Jian He [1], Jiliang Mu [1], Wenping Geng [1], Haoran Cui [1], Rui Cheng [3] and Xiujian Chou [1]

1. Science and Technology on Electronic Test and Measurement Laboratory, North University of China, Taiyuan 030051, China;
2. Fifth Clinical Medical School, Shanxi Medical University, Taiyuan 030012, China;
3. Department of Neurosurgery, Shanxi Provincial People's Hospital, Taiyuan 030012, China.

1.The following six tables show the activation values of the robotic brain regions tested in six rats.

Table S1. Stimulation parameters in S-1 rat

S-1				
		Amplitude (V)	Frequency (Hz)	Duration (S)
Left SIBF	sine	3.9	220	0.6
	triangle	4.2	250	0.8
	square	2.7	180	0.6
Left MFB	sine	3.3	180	0.7
	triangle	3.6	200	0.8
	square	2.1	130	0.6
Right MFB	sine	3	190	0.5
	triangle	3	170	0.6
	square	1.8	130	0.5
Right SIBF	sine	4.2	230	0.8
	triangle	4.5	250	0.8
	square	3.6	220	0.7

Table S2. Stimulation parameters in S-2 rat

S-2				
		Amplitude (V)	Frequency (Hz)	Duration (S)
Left SIBF	sine	5.4	250	0.7
	triangle	6	250	0.7
	square	4.2	250	0.7
Left MFB	sine	2.4	200	0.5
	triangle	2.4	200	0.7
	square	1.5	150	0.5
Right MFB	sine	2.7	200	0.6
	triangle	3	170	0.6
	square	2.1	170	0.5
Right SIBF	sine	4.2	200	0.7
	triangle	4.8	250	0.7
	square	3	200	0.7

Table S3. Stimulation parameters in S-3 rat

S-3				
		Amplitude (V)	Frequency (Hz)	Duration (S)
Left SIBF	sine	2.1	150	0.5
	triangle	2.7	180	0.6
	square	1.5	150	0.5
Left MFB	sine	2.4	180	0.5
	triangle	2.4	190	0.5
	square	1.8	150	0.4
Right MFB	sine	3	180	0.6
	triangle	3.3	180	0.6
	square	2.4	170	0.6
Right SIBF	sine	2.1	140	0.3
	triangle	2.7	150	0.3
	square	1.5	110	0.3

Table S4. Stimulation parameters in S-4 rat

S-4				
		Amplitude (V)	Frequency (Hz)	Duration (S)
Left SIBF	sine	3.6	220	0.6
	triangle	3.9	220	0.6
	square	2.7	170	0.4
Left MFB	sine	2.4	160	0.6
	triangle	2.7	170	0.6
	square	1.8	130	0.5
Right MFB	sine	2.4	170	0.5
	triangle	2.7	160	0.6
	square	1.8	120	0.4
Right SIBF	sine	4.5	240	0.7
	triangle	5.1	250	0.8
	square	3.6	200	0.4

Table S5. Stimulation parameters in S-5 rat

S-5				
		Amplitude (V)	Frequency (Hz)	Duration (S)
Left SIBF	sine	4.5	200	0.8
	triangle	4.2	240	0.7
	square	3	250	0.7
Left MFB	sine	1.8	200	0.5
	triangle	1.2	150	0.5
	square	0.6	150	0.5
Right MFB	sine	3.3	200	0.7
	triangle	2.4	150	0.7
	square	1.2	150	0.5
Right SIBF	sine	4.8	250	0.7
	triangle	4.5	250	0.5
	square	3.3	200	0.7

Table S6. Stimulation parameters in S-6 rat

S-6				
		Amplitude (V)	Frequency (Hz)	Duration (S)
Left SIBF	sine	4..8	280	0.6
	triangle	5.1	270	0.7
	square	4.2	230	0.7
Left MFB	sine	2.7	180	0.6
	triangle	2.7	200	0.6
	square	2.1	180	0.5
Right MFB	sine	2.1	180	0.6
	triangle	2.4	180	0.7
	square	1.8	150	0.5
Right SIBF	sine	4.2	230	0.6
	triangle	4.8	280	0.8
	square	3.6	250	0.7

2. Local enlargement of staining results of rat coronal brain sections.

As can be seen in the red circle in Figure S1, some neurons around the electrode paths were disarranged, and some inflammatory cells were present.

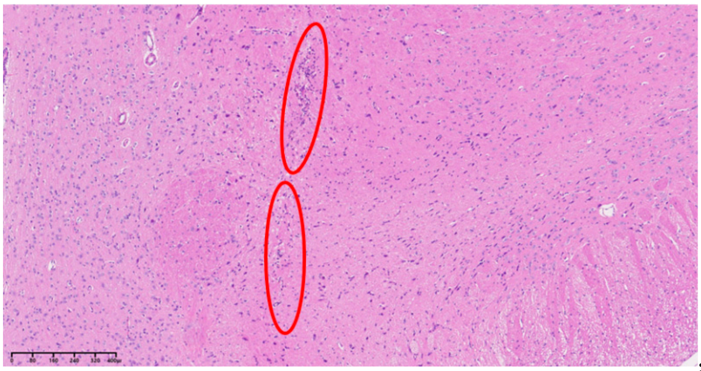,

Figure S1 Local enlargement of staining results of rat coronal brain sections.
